# Neurological adverse events associated with PD-1/PD-L1 immune checkpoint inhibitors

**DOI:** 10.3389/fnins.2023.1227049

**Published:** 2023-06-29

**Authors:** Yanting Zhou, Hongyan Li

**Affiliations:** General Surgery Department, Xuanwu Hospital, Beijing, China

**Keywords:** PD-1, neurotoxin, ICIs, adverse events, immunotherapy

## Abstract

Immunotherapy is a promising method for cancer treatment. Among them, immune checkpoint inhibitors targeting PD-1/PD-L1 are increasingly used for certain cancers. However, with the widespread use of such drugs, reports of immune-related adverse events (irAEs) are also increasing. Neurological adverse events (nAEs) are one of the irAEs that affect the peripheral and central nervous systems. They are characterized by low incidence, hard to diagnose, and life-threatening risks, which have a significant impact on the prognosis of patients. Biomarker-based early diagnosis and subsequent treatment strategies are worthy of attention, and comprehensive management of irAEs is important for optimizing patients’ quality of life and long-term outcomes. In this review, we summarized the mechanisms, common symptoms, early biomarkers, treatments, and future research directions of nAEs, in order to provide a comprehensive overview of immune checkpoint inhibitor-related nAEs targeting PD-1/PD-L1.

## 1. Introduction

Immune checkpoint inhibitors (ICIs) are a class of novel immunotherapeutic agents that are effective in treating various types of cancers. These drugs target immune checkpoint pathways to counteract immune evasion and induce anti-tumor immune responses in the body. Most ICIs that have been approved for clinical use target either cytotoxic T-lymphocyte-associated protein 4 (CTLA-4) or programmed cell death receptor-1 (PD-1) signaling. While the first approved drug, ipilimumab, is an anti-CTLA-4 antibody, monoclonal antibody drugs targeting PD-1/PD-L1 appear to be more promising due to their satisfactory therapeutic efficacy for different types of tumors, treatment regimens, drug combinations, and treatment protocols (Chen et al., 2021).

As the use of PD-1/PD-L1 inhibitors in cancer patients increases, the safety of these drugs and their associated immune-related adverse events (irAEs) have become a concern. Due to their immunological mechanisms, these drugs can cause a wide range of adverse reactions affecting various organs. Reported affected organs include the heart, skin, endocrine system, gastrointestinal tract, liver, kidneys, respiratory system, blood system, muscles, nerves, and eyes (Ramos-Casals et al., 2020). Although the safety of PD-1 inhibitors was higher than that of CTLA-4 inhibitors according to a meta-analysis of 36 phase II/III randomized trials (Xu et al., 2018), the irAEs such as hypothyroidism, pneumonia, colitis, and hypophysitis have been found to increase with the use of anti-PD-1 drugs compared with standard treatment (Baxi et al., 2018). Moreover, different PD-1/PD-L1 inhibitors appear to be associated with distinct treatment-related adverse events. Another study of 20,128 patients showed that PD-1 inhibitors are more correlated with grade 3 or higher irAEs compared to PD-L1 inhibitors (Wang et al., 2019).

Although the incidence of nAEs is low, they are difficult-to-diagnose, life-threatening and may cause permanent nerve damage (Rajendram et al., 2021). Therefore, early recognition of nAEs and appropriate interventions will undoubtedly benefit patients. To gain a more comprehensive understanding of PD-1/PD-L1-related nAEs over the past decades, we conducted a search on databases such as PubMed and Google Scholar to identify and collect relevant studies. Based on the retrieved literature, this article briefly reviews commonly used drugs targeting PD-1/PD-L1, related nAEs, early biomarkers, treatment regimens, and future research directions, in order to provide a comprehensive overview of PD-1/PD-L1 immune checkpoint inhibitor-related nAEs.

## 2. Mechanisms

### 2.1. Mechanisms of action

PD-1 is a cell surface receptor belonging to the CD28 immunoglobulin superfamily, which is usually expressed in T cells, B cells, tumor-infiltrating lymphocytes, monocytes, and dendritic cells (DC). PD-L1 is a type I transmembrane glycoprotein of the B7 ligand family that is mainly present in antigen-presenting cells (APCs). Early preclinical evidence suggests that activation of the PD-1/PD-L1 signaling pathway may be one of the mechanisms by which tumors evade antigen-specific T cell immune responses (Iwai et al., 2002).

In short, PD-L1 is expressed in some tumor cells. When tumor cells recognize the PD-1 protein on T cells, PD-L1 protein is upregulated, causing T cell apoptosis, while reducing cytokine including tumor necrosis factor (TNF), IFN-γ, and IL-2, inhibiting the function of T cells and subsequently producing immune escape effects (Daassi et al., 2020).

The binding of PD-1 to PD-L1 can transmit inhibitory signals, reduce the proliferation of CD8^+^ T cells (Nishimura et al., 2001). Therefore, researchers have proposed the possibility of predicting treatment outcomes by monitoring the proliferation of CD8^+^ T cells during targeted PD-1 pathway therapy (Kamphorst et al., 2017). A specific subset of PD-1^+^ CD8^+^ T cells was found to be proliferative burst after PD-1 blockade and the transcription factor TCF1 may plays a cell intrinsic and essential role in this process proposed by Im et al. (2016), providing a better understanding of T cell exhaustion in PD-1-directed immunotherapy.

The interactions between PD-1 and PD-L1 can also upregulate the expression of the Bcl-2 gene to control the accumulation of antigen-specific T cells in lymph nodes, thereby suppressing the immune activity of the body (Keir et al., 2005). Experiments have shown that tumor-infiltrating lymphocytes may drive the expression of the PD-1 pathway in tumor cells by secreting cytokines such as interferon-γ (IFN-γ), IL-2, IL-7, and IL-15, triggering the body’s self-immune suppression and reducing the overall survival of patients (Taube et al., 2012).

Based on this mechanism, a class of immune checkpoint inhibitors targeting PD-1/PD-L1has emerged. This type of drugs mainly consists of monoclonal antibodies, which maintain active immune responses against tumor cells by inhibiting the immune checkpoint pathway and triggering the body’s own anti-tumor immune response. The commonly used PD-1/PD-L1 immune checkpoint inhibitors in clinical practice are listed in the Table 1 below.

**Table 1:** Common PD-1/PD-L1 immune checkpoint inhibitors and the applications to cancer types approved by Food and Drug Administration (FDA).

Name	Cancer type(Food and Drug Administration (FDA) approved)
Anti-PD-1 antibodies	
Nivolumab	Non-Small Cell Lung Cancer (NSCLC) Melanoma Malignant Pleural Mesothelioma Renal Cell Carcinoma (RCC) Classical Hodgkin Lymphoma (cHL) Squamous Cell Carcinoma of the Head and Neck (SCCHN) Urothelial Carcinoma Colorectal cancer Hepatocellular Carcinoma (HCC) Gastric Cancer, Gastroesophageal Junction Cancer, and Esophageal Adenocarcinoma
Pembrolizumab	Melanoma Non-Small Cell Lung Cancer (NSCLC) Head and Neck Squamous Cell Cancer (HNSCC) Classical Hodgkin Lymphoma (cHL) Primary Mediastinal Large B-Cell Lymphoma (PMBCL) Urothelial Carcinoma Microsatellite Instability-High (MSI-H) or Mismatch Repair Deficient (dMMR) Cancer MSI-H or dMMR Colorectal Cancer (CRC) Gastric Cancer Esophageal Cancer Cervical Cancer Hepatocellular Carcinoma (HCC) Merkel Cell Carcinoma (MCC) Renal Cell Carcinoma (RCC) Endometrial Carcinoma Tumor Mutational Burden-High (TMB-H) Cancer Cutaneous Squamous Cell Carcinoma (cSCC) Triple-Negative Breast Cancer (TNBC)
Cemiplimab-rwlc	Cutaneous Squamous Cell Carcinoma (CSCC) Basal Cell Carcinoma (BCC) Non-Small Cell Lung Cancer (NSCLC)
Dostarlimab-gxly	dMMR Endometrial Carcinoma dMMR Advanced Solid Tumor
Retifanlimab-dlwr	Merkel cell carcinoma
Anti-PD-L1 antibodies	
Atezolizumab	Non-Small Cell Lung Cancer (NSCLC) Small Cell Lung Cancer (SCLC) Hepatocellular Carcinoma (HCC) Melanoma Alveolar Soft Part Sarcoma (ASPS)
Avelumab	Merkel Cell Carcinoma (MCC) Renal Cell Carcinoma (RCC) Urothelial Carcinoma (UC)
Durvalum	Non-Small Cell Lung Cancer (NSCLC) Small Cell Lung Cancer (SCLC) Biliary Tract Cancer (BTC) Hepatocellular Carcinoma (HCC)

### 2.2. Mechanisms of resistance

In the clinical use of PD-1 and PD-L1 inhibitors, it has been found that some patients can achieve long-term therapeutic effects, but the high incidence of primary resistance and acquired resistance in more patients in later stages limits their clinical application and patient prognosis, which cannot be ignored and has become a new problem urgently to be solved in this field (Lei et al., 2020).

Scholars have conducted related researches in this area. Danie et al. found through tumor biopsies of melanoma and colon cancer patients that JAK1/2 mutations in cancer cells, which will lead to unable to respond to IFN-γ by expressing PD-L1 and some other genes stimulated by interferon, resulting in a genetic mechanism that lacked reactive PD-L1 expression. Therefore, patients with such tumors are unlikely to respond to PD-1 immunotherapy (Shin et al., 2017). In addition, related mechanisms such as insufficient or lost tumor-specific antigens, MHC functional disorders, immunesuppressive microenvironments (Nowicki et al., 2018), and some other carcinogenic signaling pathways (Azuma et al., 2014) have been found to be associated with primary resistance to PD-1/PD-L1 inhibitors (Yi et al., 2022). As for acquired resistance, compensatory upregulation of inhibitory signals (Theivanthiran et al., 2020) and re-exhaustion of induced T cells (Pauken et al., 2016) can both weaken the persistence of related therapeutic effects. Therefore, combination therapy should be considered in the treatment strategy (Yi et al., 2022), including PD-1/PD-L1 inhibitors plus chemotherapy, radiotherapy, angiogenesis inhibitors, targeted therapy, other immune checkpoint inhibitors, agonists of the co-stimulatory molecule, stimulator of interferon genes agonists, fecal microbiota transplantation, epigenetic modulators, or metabolic modulators, which have already shown some potential in improving the tumor response rate and increase patient survival in existing studies (Allen et al., 2017; Gandhi et al., 2018; Kordbacheh et al., 2018; Larkin et al., 2019; Brufsky et al., 2021; Fan et al., 2021).

### 2.3. Mechanisms of neurotoxicity

Mechanisms related to neurotoxicity caused by PD-1/PD-L1 inhibitors are still limited in current research. Most researchers believe that it is mainly related to the patient’s own immune system being overactivated. Starting from previous animal studies on the PD-1/PD-L1 pathway and the mechanism of other systemic adverse reactions, perhaps the research direction of nAEs mechanism in the future can be glimpsed.

Cytokine-mediated inflammatory reactions are closely related to neurotoxicity caused by PD-1 inhibitors. Animal experiments have shown that the PD-1 signal is one of the main protective pathways that limit inflammation in the central nervous system. This inhibitory loop may reduce the release of neurotoxic factors mediated by Toll-like receptors 2 and 4 in central nervous system microglia, thus playing a crucial role in anti-inflammation. Immune therapy may also inhibit this protective pathway while producing therapeutic effects (Ren et al., 2011). Gao et al. (2022) likened PD-L1^+^ astrocytes to the gatekeepers of the brain, which can control the neuroimmune and neuroinflammatory responses related to traumatic brain injury (TBI), counteract neuroinflammation, and improve neuronal damage after traumatic brain injury. ICIs have a significant impact on the plasma levels of cytokines (Das et al., 2015). Clinically, Lim et al. (2019) have identified 11 cytokines including IL-1a, IL-2 and IFN-α2 to be significantly upregulated at baseline and early stage of treatment, integrating them into in a toxicity score (CYTOX) to predict serious adverse effects.

The development of nAE may be partially attributed to the systemic depletion of regulatory T cells (Tregs). PD-1 is not only expressed on T cells, but other immune cells and tissues are also affected by the PD-1 pathway inhibition, such as Tregs, which are direct targets of ICI immunotherapy (Yang et al., 2021). Tregs can inhibit excessive activation of the immune system, preventing and reducing neuroinflammatory autoimmune diseases of the nervous system, such as multiple sclerosis and autoimmune encephalomyelitis.

B cells are also a critical participant in autoimmune responses. Charabi et al. (2021) reported a patient with widespread transverse myelitis in whom novel neuronal autoantibodies and highly elevated CXCL13 were found in the cerebrospinal fluid, indicating that severe nAEs are mediated by B cells, which differs from the commonly accepted T-cell toxicity induced by PD-1 inhibitors. A prospective German cohort also reported the production of autoantibodies in patients who experienced neurological adverse reactions (Müller-Jensen et al., 2023), thus revalidating the role of B cells in autoimmune activation.

**Figure 1:**
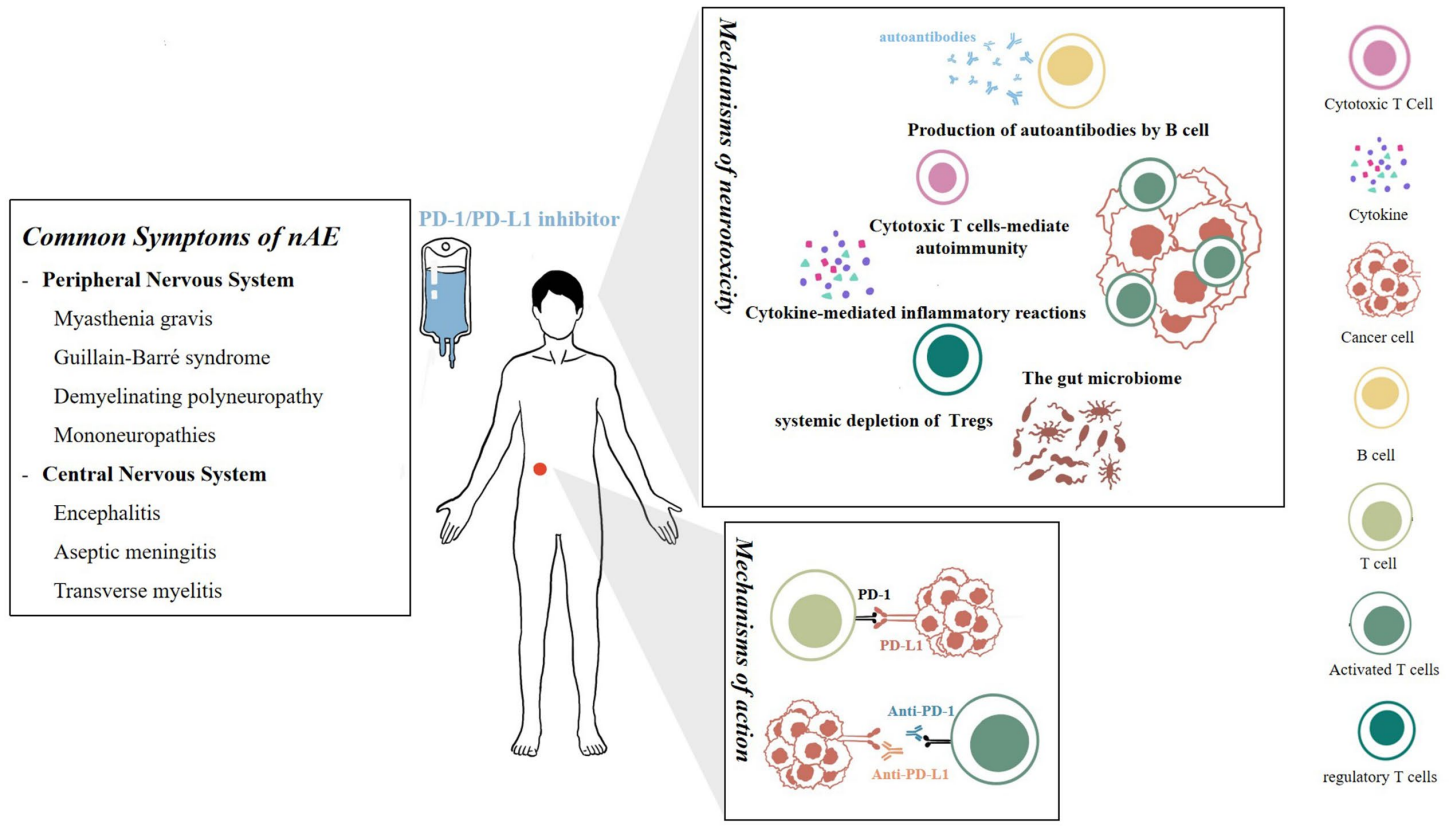
Mechanisms of action and neurotoxicity of PD-1/PD-L1 inhibitors. On left is a summary of common symptoms of nAEs.

The gut microbiome is a recently proposed mechanism theory. Sivan et al. (2015) verified in a mouse model that the microbial population can independently affect antitumor immunity, and combined immunotherapy is effective. Spencer et al. (2021) further found that low-fiber diets or probiotics in mice reduce the therapeutic response to PD-1 treatment. Relevant clinical research is also being conducted simultaneously. Derosa et al. (2018) found that antibiotics can lead to poorer progress free survival (PFS) and overall survival (OS) in patients treated with immune checkpoint inhibitors, indirectly confirming the potential regulatory ability of the gut microbiome on therapeutic efficacy. Research on the mechanisms of the gut microbiome in exploring neurotoxicity reactions is still lacking (Figure 1).

## 3. Common symptoms of nAE

PD-1/PD-L1 inhibitors have shown a good efficacy in cancer patients, becoming an adjuvant treatment and first-line treatment for many types of cancers. However, irAEs involving various systems are still observed during treatment. Although the incidence of nAE is low, they are highly heterogeneous and have an extremely wide range of effects, possibly involving various regions of the central and peripheral nervous system. Neurotoxicity is particularly prone to become chronic and has a negative impact on long-term prognosis of patients (Patrinely et al., 2021). In a study of 9,208 patients exposed to immune checkpoint inhibitors, the overall incidence of any grade nAEs with anti-PD1 antibodies was 6.1%, higher than with anti-CTLA4 antibodies (Cuzzubbo et al., 2017). Furthermore, there are different results regarding the correlation between the incidence of nAEs and the dosage of different targeted PD-1 drugs.

### 3.1. Peripheral nervous system

Myasthenia gravis is an autoimmune disease of the neuromuscular junction, characterized by fluctuating muscle weakness affecting different combinations of ocular, medullary, limb, and respiratory muscles. Compared to ipilimumab, anti-PD-1 immunotherapy is associated with more neuromuscular complications (Ruggiero et al., 2022). Studies have shown an association between myasthenia gravis and anti-PD-1 treatment (Johnson et al., 2019). Cases of anti-PD-1-induced myasthenia gravis have also been reported in recent years (Polat and Donofrio, 2016; Zhu and Li, 2016). Compared to other nAEs, it has a clinical course characterized by a high mortality rate, early onset, and frequent concomitant myocarditis and myositis (Ruggiero et al., 2022). In addition to steroids, treatment for myasthenia gravis can also consider acetylcholinesterase inhibitors such as neostigmine or pyridostigmine. Current literature is primarily based on case reports and retrospective cohort studies, with a low level of evidence, requiring mechanistic studies and prospective interventions for confirmation (Seligman et al., 2022). Additionally, Guillain-Barré syndrome (Kao et al., 2018; Rajendram et al., 2021), chronic inflammatory demyelinating polyneuropathy, and mononeuropathies (Larkin et al., 2017; Ruggiero et al., 2023) have also been reported in clinical applications.

### 3.2. Central nervous system

Encephalitis is a common adverse reaction of the nervous system and has been reported to be associated with PD-1 inhibitor immunotherapy in the clinic (Williams et al., 2016). Patients often have elevated levels of autoantibodies, but lack specificity, such as Anti-Hu (Papadopoulos et al., 2018), Anti-PCA-2 (Segal et al., 2022), Anti-GAD65 (Chung et al., 2020; Zhou et al., 2022) and Anti-Glial fibrillary acidic protein (GFAP; Kapadia et al., 2020). When diagnosing CNS encephalitis, it is necessary to differentiate it from paraneoplastic neurological diseases (PNS) by excluding infectious diseases, tumor metastasis, etc., and distinguishing it based on the time of onset of encephalitis symptoms, whether they appear after PD-1 treatment, and the effectiveness of immunosuppressive therapy. Adverse symptoms of PNS patients receiving anti-PD-1/PD-L1 immunotherapy are more likely to worsen and flare up, requiring close monitoring (Manson et al., 2019).

The incidence of aseptic meningitis is slightly lower than that of encephalitis patients (Cuzzubbo et al., 2017), and it often presents with nonspecific symptoms such as headache, fever, vomiting, and neck stiffness, with or without encephalitis. When making a differential diagnosis, it is necessary to check whether the patient’s cerebrospinal fluid is aseptic and exclude infectious diseases. Specific treatment methods are described in detail below.

Transverse myelitis is a rare complication of the nervous system that often appears 4 weeks after administration. Most reports of transverse myelitis are related to Pembrolizumab (Wilson et al., 2018; Charabi et al., 2021). In addition to typical clinical symptoms, patients also show elevated levels of novel neuron autoantibodies and CXCL13, suggesting a possible B-cell-related mechanism. Conventional corticosteroid treatment and plasma exchange are clinically effective.

## 4. Biomarkers for early prediction

Considering the adverse effects of neurological reactions on the prognosis of patients, which may even lead to early termination of PD-1 targeted immunotherapy, early identification of high-risk patients for nAE is of great clinical significance to reduce or avoid severe adverse events. To achieve this goal, some biomarkers and risk factors for early prediction have been proposed to facilitate early recognition and prompt management of adverse reactions in clinical practice. The biomarkers mentioned in this review have been summarized in Table 2.

**Table 2:** The current biomarkers for early prediction of PD-1/PD-L1 inhibitors related nAEs.

Biomarkers	Author	Year	Type of study	Relationship with nAEs
C-reactive protein	Yu et al.	2022	Retrospective study	Patients with nAEs had higher levels of C-reactive protein (CRP)
IL-6	Hailemichael et al.	2022	Preclinical models	Combined IL-6 blockade and ICB enhanced tumor rejection while simultaneously mitigating EAE symptoms versus ICB alone
Autoantibodies	Ruste et al.	2021	Prospective study	Pre-existing autoimmune are significantly associated with an increased risk of irAE
	Müller-Jensen et al.	2023	Prospective study	Neuromuscular autoantibodies may serve as a feasible marker to diagnose and potentially predict life-threatening ICI-induced neuromuscular disease
Microorganisms	Liu et al.	2021	Prospective study	Five microbial biomarkers can successfully distinguish patients without irAEs from those with severe irAEs
	Liu et al.	2023	Prospective study	The gut microbiota is important in dictating irAE occurrence and type

Autoantibodies are a key biomarker that has received much attention. Data from REISAMIC (Abdel-Wahab et al., 2018) show that pre-existing autoimmune or inflammatory diseases are significantly associated with an increased risk of irAE in patients receiving anti-PD-1 antibody therapy, suggesting a possible correlation between autoantibodies and adverse reactions. Recently, researchers such as Leonie have prospectively collected relevant data from patients treated with ICIs and found that neuromuscular autoantibodies, including anti-titin, anti-skeletal muscle, anti-heart muscle, anti-LRP4, anti-RyR, and anti-AchR, can serve as feasible biomarkers for the diagnosis and potential prediction of ICI-induced peripheral neuropathies, such as myositis and severe myasthenia gravis, with a high sensitivity of 80% (95% CI 0.52–0.96) and a specificity of 88% (95% CI 0.76–0.95) (Müller-Jensen et al., 2023). Some anti-tumor antibodies have also been reported in case reports of adverse reactions (Gill et al., 2019). However, there is still controversy over whether autoantibodies should be routinely screened before treatment due to the variety of autoantibodies, the high cost, and the fact that they are not an absolute contraindication for PD-1 immunotherapy in clinical practice. It is necessary to improve the cost-effectiveness and select more autoantibodies that are most strongly associated with irAE through more large-scale, prospective data screening to identify high-risk patients precisely for prophylactic treatment.

In peripheral blood indicators, researchers often focus on related immune molecule. As a biomarker, C-reactive protein and IL-6 have the advantages of being easily obtained and low-cost in clinical practice. Yu et al. (2022) already verified their feasibility as potential biomarkers for adverse events through retrospective analysis. A new flow cytometry method based on prospective studies has been proposed, monitoring of ICI occupancy through combining measurement of anti-PD-1 occupancy and evaluation of remaining PD-1 receptor availability with anti-IgG4 PE and anti-PD-1 BV421 (Gazzano et al., 2022). The CYTOX score (Lim et al., 2019), a toxicity score, is also presented as a new method for predicting severe adverse reactions, which includes 11 cytokines such as IL-1a, IL-2 and IFNα2, found significantly upregulated in 65 cytokines that express in 98 melanoma patients and validated in a 49-person cohort. Microorganisms are also a promising research direction. Intestinal microbiota in feces is a potential biomarker for predicting the efficacy and prognosis of PD-1 inhibitors (Deluce et al., 2022). Liu et al. (2021) collected fecal samples from patients in a prospective study and found that *Streptococcus*, Paecalibacterium, and Stenotrophomonas were significantly more abundant in patients with severe irAEs, while *Faecalibacterium* and unidentified Lachnospiraceae were more abundant in patients with mild irAEs. A classification model based on five microorganisms could successfully distinguish patients without irAEs from those with severe irAEs (Liu et al., 2021). Similarly, another similar clinical trials showed that the microbiome has a significant correlation with irAEs (Liu et al., 2023). Based on this, fecal microbiota transplantation (FMT) has also been proposed as a promising treatment option for adverse reactions, and has been shown to be effective in in mouse models (Vétizou et al., 2015). This has important implications for the future of PD-1/PD-L1 immunotherapy.

## 5. Treatment

Treatment strategies which commonly used in clinical have been continuously updated as the understanding of drug mechanisms deepens. From simply reducing drug dosage or discontinuing medication, to the intervention of drugs such as steroids, immunomodulators, and monoclonal antibodies, and more recently, the exploration of traditional Chinese medicine treatment. Overall, this area is still in the exploratory stage and a standardized diagnostic and treatment plan has not yet been established. However, some scholars have proposed relevant guidelines for clinical reference (Haanen et al., 2017; Brahmer et al., 2018; Spain et al., 2019).

In principle, before conducting immunotherapy, doctors should inform patients and their families of relevant information, especially high-risk patients for adverse reactions of the nervous system, in order to detect related symptoms earlier, timely discontinue medication, and adopt effective intervention measures. Prompt examination to differentiate diagnosis is also emphasized after discovering nervous system symptoms. Specific treatment can be carried out after excluding factors such as tumor metastasis and bacterial meningitis.

Existing clinical treatment plans are mostly based on the level of patients’ adverse reactions. The most commonly used scale is the Common Terminology Criteria for Adverse Events (CTCAE) published by the National Cancer Institute (NCI) in the United States. Grade 1 usually does not require additional intervention measures, and close monitoring and temporary discontinuation of medication are the most commonly used management strategies, which can be monitored routinely by oncologists. Patients with Grade 2 or above are recommended to discontinue treatment and adopt drug interventions such as steroids, and if necessary, request consultation from specialists. The following section introduces commonly used drugs for treatment, which have been summarized in Table 3.

**Table 3:** The existing treatment options of PD-1/PD-L1 inhibitors related nAEs.

Treatment	Medicine for clinical use	Recommended level	Reference
Steroids	Prednisolone Methylprednisolone	First-line	Spain et al. (2019)
Plasma exchange and immunoglobulin injections	——	Second-line	Meregalli et al. (2021) and Touat et al. (2017)
Monoclonal antibodies	Tolimumab Rituximab	Individualized administration regimen, lacking randomized trials	Picca et al. (2021), Stroud et al. (2019), and Williams et al. (2016)
Traditional Chinese medicine (TCM)	Astragalus, ginsenoside Rg3 The compound formulae of All Nourishing Decoction Buzhong Yiqi Decoction	Individualized administration regimen, lacking randomized trials	Zhang et al. (2020) and Shao et al. (2017)

### 5.1. Steroids

Steroids are most commonly used in the cases as the first-line regimen and are the cornerstone of clinical treatment for nAEs due to PD-1/PD-L1 inhibitors. Typically, prednisolone or methylprednisolone is often chosen in corticosteroid therapy, depending on the severity of the adverse reactions, and is slowly tapered after symptom resolution and continued for at least 4 weeks. However, considering the long half-life of PD-1/PD-L1 inhibitors, the duration of steroid use can be extended appropriately.

The efficacy of steroids is positive, and patients who receive steroids at the time of initiation of PD-1 immunotherapy appear to have fewer adverse events than those who do not (Abdel-Wahab et al., 2018). However, Bai et al. (2021) found that early use of high-dose GCC was associated with poorer PFS and OS after an irAE episode, suggesting that modest use of GCC early in anti-PD-1 monotherapy should be considered. In summary, further prospective randomized controlled clinical trials are warranted to explore the best management options for irAE.

### 5.2. Plasma exchange and immunoglobulin injections

Intravenous immunoglobulin is effective in reducing neurotoxicity (Garcia et al., 2018; Meregalli et al., 2021) and is often used as second-line therapy for nAEs (Touat et al., 2017). Some nAEs are associated with autoantibodies, and treatment with plasma exchange can remove pathogenic autoantibodies from the circulation (Chen et al., 2014), which is particularly effective in severe myasthenia gravis or Guillain-Barré syndrome, and has been reported to be effective in the treatment of encephalitis (Burke et al., 2018). This therapy is commonly used in patients with severe adverse reactions to steroid therapy that is not effective.

### 5.3. Monoclonal antibodies

With the intensive research on drug mechanisms, some monoclonal antibody therapies have also been shown to be effective in practice. Tolimumab, an IL-6 monoclonal antibody, can benefit some steroid-resistant patients, especially those with a significant rise in IL-6 detected in the cerebrospinal fluid (Picca et al., 2021). In a retrospective study, 79.4% (*n* = 34) of patients treated with up to four doses of tolimumab showed clinical improvement in adverse effects (Stroud et al., 2019). Rituximab (Williams et al., 2016) has also been reported for the treatment of autoimmune encephalitis in the presence of anti-N-methyl-D-aspartate receptor antibodies in the cerebrospinal fluid. Although monoclonal antibodies are potential options for the treatment of steroid-refractory adverse reactions, no large-scale randomized trials have elucidated the relative efficacy and safety of these agents.

### 5.4. Traditional Chinese medicine

In recent years, TCM has also shown exciting possibilities in the prevention and treatment of adverse reactions to PD-1 inhibitors, with clinical data confirming that immunotherapy combined with TCM can effectively mitigate related adverse reactions (Lam et al., 2018; Liu et al., 2019).

By modulating the body’s immune function and tumor immune microenvironment, TCM is theoretically sufficient to achieve preventive mitigation of irAEs (Zhang et al., 2020). According to TCM doctors, PD-1/PD-L1 inhibitors are “warm, hot, sweet, and pungent” in Chinese medicine, with a tendency to rise and float. “Immunotoxicity” is the main etiological factor, and the main pathogenesis is characterized by wind-evil combined with dampness and heat toxicity. The strong power of warming and dispersal can easily carry wind and make fire and heat reach outside, depleting qi and injuring fluid, thus leading to inflammation related to the nervous system, so add wind-dispersing and heat-dissipating herbs, benefiting qi and nourishing yin, such as forsythia and Honeysuckle flower. In the theory of “supporting the righteous and dispelling the evil,” tonic herbs are also considered as an effective choice, such as Astragalus, ginsenoside Rg3 (Shao et al., 2017), and the compound formulae of All Nourishing Decoction (Ishikawa et al., 2017) and Buzhong Yiqi Decoction (Liu et al., 2019). Non-pharmacological approaches specific to TCM, such as acupuncture (Sun et al., 2021), have also been shown to have neuroprotective and anti-inflammatory effects, significantly improving immune function and reducing inflammatory factor levels.

Both TCM and immunotherapy are systemic and complex, and research on TCM for neurological adverse reactions is even more lacking, and today there is still no standardized management strategy. Further research and large-scale clinical trials are needed to determine the mechanisms and efficacy of TCM, so that TCM can play a greater role in clinical prevention and treatment of PD-1-induced nAEs by providing more safe and effective combination drug regimens.

## 6. Summary and outlook

In recent years, PD-1/PD-L1 inhibitors have been widely approved for use in many cancer types, and increasingly patients are benefiting from them. With the gradual increase of patient data in the clinic, the direction of related research is gradually transforming from mechanism of action to clinical use. The occurrence of nAEs in patients, although low in incidence, is often difficult to diagnose and has a poor prognosis, leading to interruption or even cessation of treatment. Therefore, separating the beneficial antitumor therapeutic activity of such drugs from the appearance of nAE is now a major challenge to achieve optimal efficacy of cancer immunotherapy.

The clinical signs of nAE can be divided into peripheral and central nervous system injuries. A number of retrospective studies and reviews with larger sample sizes are now available that summarize this more comprehensively. However, the complexity of the immune system, the low incidence of neurological adverse reactions, and the difficulty of prospectively obtaining clinical samples (e.g., antibodies and cerebrospinal fluid) have limited the progress of the studies, and it may take longer to obtain larger sample sizes of neurological adverse reaction cohorts. A definitive diagnosis is also critical to clinical data collection and determines the accuracy of morbidity. This often requires collaboration between specialty-trained oncologists and neurologists.

PD-1/PD-L1 inhibitors are often used clinically in combination, and whether the combination with pre-existing chemotherapy or certain novel targeted therapies increases the likelihood of adverse events is closely related to drug safety and is one of the directions worth investigating in the future (Schoenfeld et al., 2019). It has been shown that patients who experienced irAE performed significantly better in terms of progression-free survival, overall survival and overall remission rate compared to patients who lacked irAE (Das and Johnson, 2019). The next step might be to further explore whether there is a correlation between the incidence of neurological adverse reactions or a specific adverse reaction, such as the occurrence of encephalitis or myasthenia gravis, and efficacy.

With regard to biomarkers, in addition to in-depth research in existing directions, the use of artificial intelligence and big data in may also be a feasible direction for subsequent research, which is to combine large-scale retrospective data for analysis to obtain relevant and feasible biomarkers or independent risk factors, aiming to construct a predictive model for adverse neurological effects. Aberrations in brain MRI after ICI treatment have also been found, and the performance varies greatly among patients, but patients with brain MRI aberrations tend to have a better prognosis (Ni et al., 2022), and prediction based on this combined with imaging changes is also a future direction. In addition, gender differences in nAEs have been identified, but basic studies are still lacking.

For the management of patients with nAEs, most of the existing protocols are individualized with symptomatic treatment, and there is still a lack of standardization for the determination of discontinuation and indication of drug use. With the widespread use of PD-1 in cancer patients, the clinical sample size for the emergence of nAEs will also increase, which will facilitate the improvement and establishment of clinical management protocols in the coming years. The impact of existing treatment regimens on drug efficacy is also a direction worth exploring.

PD-1/PD-L1 inhibitors have transformed clinical cancer treatment but have also led to immune-related neurotoxicity. Improving the safety of this kind of drugs is an important issue to be urgently addressed and expanding the population benefiting from PD-1 immunotherapy so that more patients can reap better outcomes and prognosis is a common vision of physicians and scientists. Collaboration in different fields is called for, whether it is the exchange between clinical and basic research or the formation of cancer patient management teams by physicians from different specialties, such as neurologists, oncologists, and radiologist, as the future direction of cancer treatment.

## Author contributions

YZ and HL conceived and wrote the manuscript and helped to develop all tables and the figure. HL read and approved the final version of the manuscript. All authors contributed to the article and approved the submitted version.

## Funding

This work was supported by the National Natural Science Foundation of China (No. 82271373), R&D Program of Beijing Municipal Education Commission (KM202110025019), and Natural Science Foundation of Beijing Municipality (No. 7232075).

## Conflict of interest

The authors declare that the research was conducted in the absence of any commercial or financial relationships that could be construed as a potential conflict of interest.

## Publisher’s note

All claims expressed in this article are solely those of the authors and do not necessarily represent those of their affiliated organizations, or those of the publisher, the editors and the reviewers. Any product that may be evaluated in this article, or claim that may be made by its manufacturer, is not guaranteed or endorsed by the publisher.
